# Colchicine therapy in children with FMF

**DOI:** 10.1186/1546-0096-13-S1-O44

**Published:** 2015-09-28

**Authors:** A-M Knieper, J Klotsche, D Föll, H Wittkowski, E Lainka, T Kallinich

**Affiliations:** 1 Charité - Universitätsmedizin Berlin, Klinik für Pädiatrie mit Schwerpunkt Pneumologie und Immunologie, Berlin, Germany; 2 DRFZ Berlin - Deutsches Rheuma-Forschungszentrum, Berlin, Germany; 3 Universitätsklinikum Münster, Institut für Immunologie, Münster, Germany; 4 Universität Duisburg-Essen, Uniklinikum Essen, Kinderklinik, Pädiatrische Rheumatologie, Essen, Germany

## Introduction

Colchicine treatment is the standard therapy for prophylaxis of attacks and amyloid deposition in familial Mediterranean fever. In the guidelines of 2007 an initial dosage in regard of the age of the patient is recommended (0.5 mg/day for children <5 years of age, 1.0 mg/day for children 5-10 years of age, 1.5 mg/day for children >10 years of age).

## Objectives

The following objective were raised in the study: To analyze factors, which influences the individual colchicine dose in children with FMF. To analyze the impact of dose adjustment on the clinical course and the degree of subclinical inflammation. To analyze parameters, which predict dose increase in the upcoming 12 month including levels of SAA and S100A12-molecules

## Patients and methods

Data of the FMF-patients were extracted from the AID-Net-register and analysed with SPSS (Patient number n=409, Visit number n= 4750). Correlation and regression analyses were performed with the aim of an multivariant analyses. The extraction of the data was in January 2015.

## Results

Colchicine dose correlates with the genotype. It further increases linearly with an increase of age (1 - 18 years), body weight and body surface area. The correlation of colchicine dose with body surface revealed an average of 1 mg colchicine / 1 m^2^.

A high response rate in the CRP and the attack rate is shown both at time of therapy introduction (70% response CRP: 45.5% / attack frequency: 61.4%), as well as dose increases during the course of the disease (with an increase of 1.0 mg to 1 5 mg: 70% Response CRP: 32.1% / attack frequency: 42.3%).

The severity of disease at diagnosis increases the likelihood of a dose raise in the first 12 months and 24 months after therapy introduction.

An increase of classical inflammatory markers (CRP, SAA) and S100 proteins show a significant correlation to a dose increase in the following 12 or 24 months (p <0.05).

## Conclusion

The mutation, the age, the body weight, the body surface area and the initial disease severity affect the colchicine dose.

Dose increase is effective to control disease activity and subclinical inflammation. This effect was also observed when increasing the dose from 1.5 mg to 2.0 mg a day.

An increase in the classical inflammation markers and S100A12- or S100A8 / A9-value are predictors for an upcoming increase in the dose in the next 12 or 24 months, None of these factors showed an advantage in predicting a higher dose.

